# Downregulation of CISD2 Has Prognostic Value in Non-Small Cell Lung Cancer and Inhibits the Tumorigenesis by Inducing Mitochondrial Dysfunction

**DOI:** 10.3389/fonc.2020.595524

**Published:** 2021-02-01

**Authors:** Fangchun Shao, Yanchun Li, Wanye Hu, Jiaqi Yu, HengYu Wu, Kejing Ying, Jun Xia, Jing Du

**Affiliations:** ^1^Department of Respiratory, Zhejiang Provincial People’s Hospital, People’s Hospital of Hangzhou Medical College, Hangzhou, China; ^2^Department of Laboratory Medicine, Zhejiang Provincial People’s Hospital, People’s Hospital of Hangzhou Medical College, Hangzhou, China; ^3^Department of Respiratory, Sir Run Run Shaw Hospital, School of Medicine, Zhejiang University, Hangzhou, China

**Keywords:** CISD2, iron-sulfur cluster, mitochondria, oxidative stress, iron metabolism

## Abstract

CISD2, a NEET protein that coordinates 2Fe-2S clusters through its CDGSH domain, is critical for normal development and iron homeostasis. CISD2 plays an important role in Fe-S cluster transfer and promotes cancer proliferation. However, its specific role in the development of non-small cell lung cancer (NSCLC) remains unclear. Bioinformatics of pan-cancer analysis from The Cancer Genome Atlas show that CISD2 has an aberrant expression in most types of human cancers. Moreover, CISD2 expression is associated with a higher hazard ratio and exhibits significantly poorer overall survival in lung adenocarcinoma (LUAD), uveal melanoma, head and neck squamous cell carcinoma, brain lower grade glioma, kidney chromophobe, and liver hepatocellular carcinoma. Further investigation revealed that CISD2 is highly expressed in LUAD and LUSC, which is associated with clinical pathological stages. In addition, survival data collected from GSE31210 and GSE13213, two datasets from the NCBI Gene Expression Omnibus, also confirmed that high CISD2 expression is associated with unfavorable survival in patients with LUAD. A cell-based assay indicated that the knockdown of CISD2 inhibited proliferation, invasion, and migration in A549 cells. Additionally, CISD2 knockdown accelerated the accumulation of cellular and mitochondrial reactive oxygen species, destroying the mitochondrial morphology and function. Moreover, CISD2 inhibition activated the iron starvation response, thus, accelerating iron accumulation in A549 cells. Pretreatment with DFO, the iron chelator, blocked mitochondrial dysfunction in CISD2-knockdown cells. Collectively, the present study provides novel insights into the regulatory role of CISD2 in NSCLC and presents a potential target to improve antitumor activity based on oxidative stress.

## Introduction

Lung cancer is one of the malignant tumors with the fastest increasing morbidity and mortality rates and is currently the greatest threat to people’s health and life globally. In 2020, an estimated 135,000 deaths from lung cancer have occurred so far in the USA (1). In China, lung cancer ranks first in the incidence of malignant tumors, with approximately 787,000 new cases per year, which accounts for 20.03% of all new cancer cases. For deaths due to cancer, lung cancer also ranks first (26.99%), with 631,000 deaths per year (2). As the major histological type, non-small cell lung cancer (NSCLC) accounts for ~80% of all lung cancer cases, in which lung adenocarcinoma (LUAD) accounts for ~50% (3). The average five-year survival rate is only 17.4%. At present, the median survival time of NSCLC in developed countries remains less than 1.5 years. Given such high mortality rates, it is crucial that we improve our understanding of the occurrence and progression of NSCLC to develop new therapy targets for improved treatment options.

The highly conserved NEET family comprises a unique class of iron-sulfur (Fe-S) proteins that harbor the 3Cys-1His [2Fe-2S]-binding CDGSH domain, which is important in human health and disease (4). The NEET family proteins are unique because their [2Fe-2S] cluster is both redox-active and labile by binding with the CDGSH motif, in which the His amino acid residue is solvent-accessible and can be easily protonated (5, 6). In humans, only three different genes are currently known to encode for NEET proteins. *CISD1*, also known as mitoNEET, codes for a homodimeric protein that is localized to the outer mitochondrial membrane and participates in oxidation regulation. *CISD3*, also known as Miner2 or MiNT, differs from the other family members because it encodes a monomer containing two CDGSH motifs. Current research suggested that it coordinates a complementary role in mitochondrial iron and reactive oxygen species (ROS) regulation within the mitochondrial matrix (7). *CISD2* encodes for NAF-1 (CISD2), which is localized to the outer mitochondrial membrane and endoplasmic reticulum, and is primarily involved in autophagy regulation, Ca^2+^ homeostasis, and mammalian lifespan control. CISD2 has an aberrant expression in several types of human cancers. However, its role in tumorigenesis and the underlying mechanism require further study.

In this study, based on the analysis of public database, we found that CISD2 has an aberrant expression in most types of human cancers and is associated with poorer overall survival (OS) for NSCLC. A cell-based assay indicated that the knockdown of CISD2 inhibited proliferation, invasion, and migration in A549 cells. Additionally, CISD2 knockdown accelerated the accumulation of cellular and mitochondrial ROS, destroying the mitochondrial morphology and function. Moreover, CISD2 inhibition activated the iron starvation response, thus, accelerating iron accumulation in A549 cells. Pretreatment with deferoxamine (DFO), the iron chelator, may block mitochondrial dysfunction in CISD2-knockdown cells. Collectively, the present study provides novel insights into the regulatory role of CISD2 in NSCLC and presents a potential target to improve antitumor activity based on oxidative stress.

## Materials and Methods

### Sample Data Sources

The data of samples analyzed in this paper were from public database, Genotype-Tissue Expression Project (GTEx) and The Cancer Genome Atlas (TCGA). GTEx portal (https://gtexportal.org/) has updated to data release V8 (dbGaP accession phs000424.v8.p2), includes genotype data from approximately 948 post-mortem donors and approximately 17,382 RNA-seq samples across 54 tissue sites and 2 cell lines. TCGA (https://www.cancer.gov/about-nci/organization/ccg/research/structural-genomics/tcga) is a landmark cancer genomics program; thus far, it has produced RNA-Seq data for 9,736 tumor samples across 33 cancer types and data for 726 adjacent normal tissues. Due to the limited normal samples from TCGA, the SangerBOX data analysis platform (http://sangerbox.com/Index) was used to integrate the data resources of the TCGA and GTEx databases to analyze the expression of CISD2.

### UALCAN Database Analysis

UALCAN, publicly available at http://ualcan.path.uab.edu, is a comprehensive and user-friendly web resource for in-depth analyzing TCGA cancer data, provides access to determine graphs and plots depicting gene expression and survival curves. In this study, we used UALCAN to analyze the gene expression of CISD2 in lung cancers based on sample types and individual cancer stages.

### PrognoScan Database Analysis

The PrognoScan database (http://dna00.bio.kyutech.ac.jp/PrognoScan/index.html), a tool for assessing the biological relationship between gene expression and prognosis, is a large collection of publicly available cancer microarray datasets with clinical annotation. PrognoScan employs the minimum P-value approach for grouping patients for survival analysis that finds the optimal cutpoint in continuous gene expression measurement without prior biological knowledge or assumption and, as a result, enables systematic meta-analysis of multiple datasets. We also used this database to assess the relationship between CISD2 expression and patient outcome in databases other than TCGA.

### The Human Protein Atlas (HPA)

The Human Protein Atlas (https://www.proteinatlas.org/) shows the expression and localization of human proteins across tissues and organs, and the representative immunohistochemistry images of CISD2 in normal lung tissue and lung cancer tissues were downloaded from HPA.

### Cell Culture

293T and NSCLC cell line A549 were obtained from The Cell Bank of Chinese Academy of Sciences (Shanghai, China) and cultured in DMEM medium (Hyclone, Logan, UT, USA) containing 10% fetal bovine serum (Gibco, Grand Island, NY, USA), 100 U/ml penicillin, and 100 μg/ml streptomycin. Cells were maintained in an incubator with a humidified atmosphere of 5% CO_2_ at 37°C and used within 20 passages.

### Antibodies and Reagents

The antibodies to CISD2 (60758 S), IRP2 (37135S), and HIF-1α (#36169) were obtained from Cell Signaling Technology (Danvers, MA, USA). The antibodies to Transferrin Receptor (sc-32272) were obtained from Santa Cruz (Dallas, TX, USA). The antibodies to β-Actin (ab8226) were obtained from Abcam (Cambridge, MA, USA). CCK-8 Assay Kit was obtained from Meilunbio (Dalian, China). C11-BODIPY (581/591), deferoxamine (DFO), Rhodamine B-[(1, 10-phenanthroline-5-yl)-aminocarbonyl] benzyl ester (RPA), DCF-DA, MitoTracker, and MitoSOX were obtained from Thermo Fisher Scientific (Waltham, MA, USA). The BeyoClick™ EdU Cell Proliferation Kit and ATP detection kit purchased from Beyotime (Shanghai, China), Annexin V-FITC/PI apoptosis detection kit was purchased from TransGen Biotech (Beijing, China).

### Construction of Expression Vector for CISD2 Knockdown

For knockdown of CISD2, target shRNA sequences were subcloned into pLVX-shRNA lentivirus vector (Takara). The shRNA sequences for CISD2 knockdown were shown as below. shCISD2-1 forward primer: GATCCGGATAGCTTGATTAATCTTAATTCAAGAGATTAAGATTAATCAAGCTATCCTTTTTTG, shCISD2-1 reverse primer: AATTCAAAAAAGGATAGCTTGATTAATCTTAATCTCTTGAATTAAGATTAATCAAGCTATCCG, shCISD2-2 forward primer: GATCCGAGATAATGTGGGTCCACTAATTTCAAGAGAATTAGTGGACCCACATTATCTTTTTTTG. shCISD2-2 reverse primer: AATTCAAAAAAAGATAATGTGGGTCCACTAATTCTCTTGAAATTAGTGGACCCACATTATCTCG. The recombinant lentiviral plasmids were purified by an extraction kit (Accurate Biotechnology, Hunan) and verified by sequencing. The vector with a scramble DNA was used as a control.

### Lentivirus Package

293T cells were used for packaging lentivirus. Briefly, the recombinant pLVX-shRNA lentiviral plasmid, pMD2G and pSPAX2 were co-transfected into 293T cells according to the instructions of lip3000 transfection kit to produce recombinant lentivirus for silencing CISD2.

### Knockdown of CISD2

A549 cells were seeded in six-well plates (NEST Biotechnology) and transfected with scramble or CISD2 knockdown lentivirus with an MOI of 20, respectively. At 24 h post-infection, the cells were collected by centrifugation and suspended with warmed complete culture medium for another 24 h and followed by selective antibiotics. After screening for 7 days, cells were harvested and the expression of CISD2 was tested by immunoblotting. The verified cells were used for subsequent experiments.

### Western Blot

Cells were harvested and washed twice with phosphate-buffered saline (PBS). Total protein extracts from cells were obtained after being lysed in a cooling RIPA lysis buffer (Beyotime, P0013B) containing 1 mM phenylmethylsufonyl fluoride (PMSF) for 15 min and centrifuged at 12,000 rpm for 5 min. The supernatant containing total protein were determined for quantitation by the BCA protein assay kit. An equal amount of samples (20 μg/lane) were loaded on SDS-PAGE and underwent electrophoresis and membrane transfer. Next, membranes were blocked and incubated with primary antibodies overnight at 4°C. After being washed with tris buffer saline containing 0.1% Tween-20 (TBST), membranes were incubated with secondary antibodies for 1 h at room temperature, then being washed again and visualized by the chemiluminescence system. Western blot data presented are representative of three independent experiments, β-Actin was used as a loading control.

### Cell Viability Assay

An equal amount of the reconstructed cells (10,000 cells for each well) were seeded in 96-well culture plates and the cell viability was evaluated according to the instruction of Cell Counting Kit-8 (CCK8) assay kit (Meilunbio, China). This kit contains the regent of WST-8, which could be reduced by cellular dehydrogenases to an orange formazan product, soluble in cell culture medium and with a specific absorption peak at 450 nm. The amount of produced formazan is directly proportional to the number of living cells.

### EdU Incorporation Assay

Cell proliferation was tested with an EdU (5-ethynyl-2’-deoxyuridine) incorporation assay, which was performed with a BeyoClick™ EdU Cell Proliferation Kit (Beyotime, C0078S) in accordance with the manufacturer’s instruction. Results were acquired using a laser confocal microscope (Leica, Germany).

### Cellular ROS Assay

Equal amounts of cells were harvested at the end of treatment and washed with phosphate-buffered saline (PBS) three times. The cell pellets were then resuspended and stained with 5 μM DCF-DA, a cell-permeable non-fluorescent probe which is de-esterified intracellularly and turns to highly fluorescent 2′,7′-dichlorofluorescein (DCF) upon oxidation. DCF-DA is useful for the detection of reactive oxygen species (ROS) and nitric oxide (•NO) and for the determination of overall oxidative stress. After incubation in the dark for 20 min at 37°C, cells were washed with PBS three times again and subsequently suspended for detection by flow cytometry.

### Mitochondrial ROS Assay

The cell pellets were harvested as usual and then resuspended and stained with MitoSOX Red reagent (3 μM), a live-cell permeant and rapidly and selectively mitochondria-targeted probe which could readily be oxidized by superoxide but not by other ROS or reactive nitrogen species (RNS) generating systems. MitoSOX may directly measure the superoxide generated in the mitochondria of live cells. After incubation in the dark for 20 min at 37°C, cells were washed with PBS three times again and subsequently suspended for detection by flow cytometry.

### Wound−Healing Assay

A549 cells with or without CISD2 knockdown were seeded into a six−well plate at a confluence of 80%. When cell confluence reached to 95%, a 100 μl pipette tip was used to scratch a straight line into the cell layer. Cells were washed with PBS for three times and incubated with serum-free medium for 36 h. Images were captured under a microscope (Nikon). The length of the wound was measured using Image J software.

### Oxygen Consumption Determination

The Seahorse XFe24 Analyzer (Agilent Technologies, Santa Clara, CA, USA) was used to measure the real-time oxygen consumption rate (OCR) of A549 cells with modified CISD2 expression according to the manufacturer’s protocol. Briefly, cells (5 × 10^4^/well) were seeded into 24-well cell plates and incubated at 37°C, 5% CO2 overnight. Meanwhile, the sensor cartridge was hydrated at 37°C in a non-CO2 incubator overnight. The next day, cells were replaced with seahorse assay medium and running the protocol until the calibration was completed. The cells were exposed to the following compounds sequentially: oligomycin (1.5 μM), FCCP (1.5 μM), rotenone (0.5 μM), and antimycin A (0.5 μM). The number of cells in each well was counted and used to normalize the data.

### Mitochondrial Morphology Analysis

Control and CISD2 silenced cells were seeded in a chamber confocal dish and treated with or without DFO (100 μM) for 12 h. Then cells were stained with MitoTracker (200 nM) in the dark for 20 min, and restained with DAPI (10 µg/ml) for another 10 min. Images were viewed and captured under a confocal microscope.

### Transmission Electron Microscope

At the end of DFO treatment, control and CISD2-silenced cells were fixed in glutaraldehyde solution for overnight at 4°C. Then, the samples were dehydrated through a graded series of ethanol, and dehydrated by alcohol and eventually transferred to absolute acetone. After infiltration with absolute acetone and the final spurr resin mixture, the samples were embedded, sectioned, stained, and finally observed and captured in the Hitachi Model H-7650 transmission electron microscope.

### Statistical Analysis

Each experiment was performed independently three times and data were shown as mean±standard deviation (SD). The Student’s t-test was used to analyze differences between two groups, and two-way ANOVA was used when more than two groups were compared. Survival curves were protracted using the Kaplan-Meier method and estimated by the log-rank test. Statistical analysis was performed using GraphPad Prism 5.0 and *P* < 0.05 was considered to be statistically significant.

## Results

### CISD2 Expression Is Upregulated in Multiple Cancers

**Figure 1:**
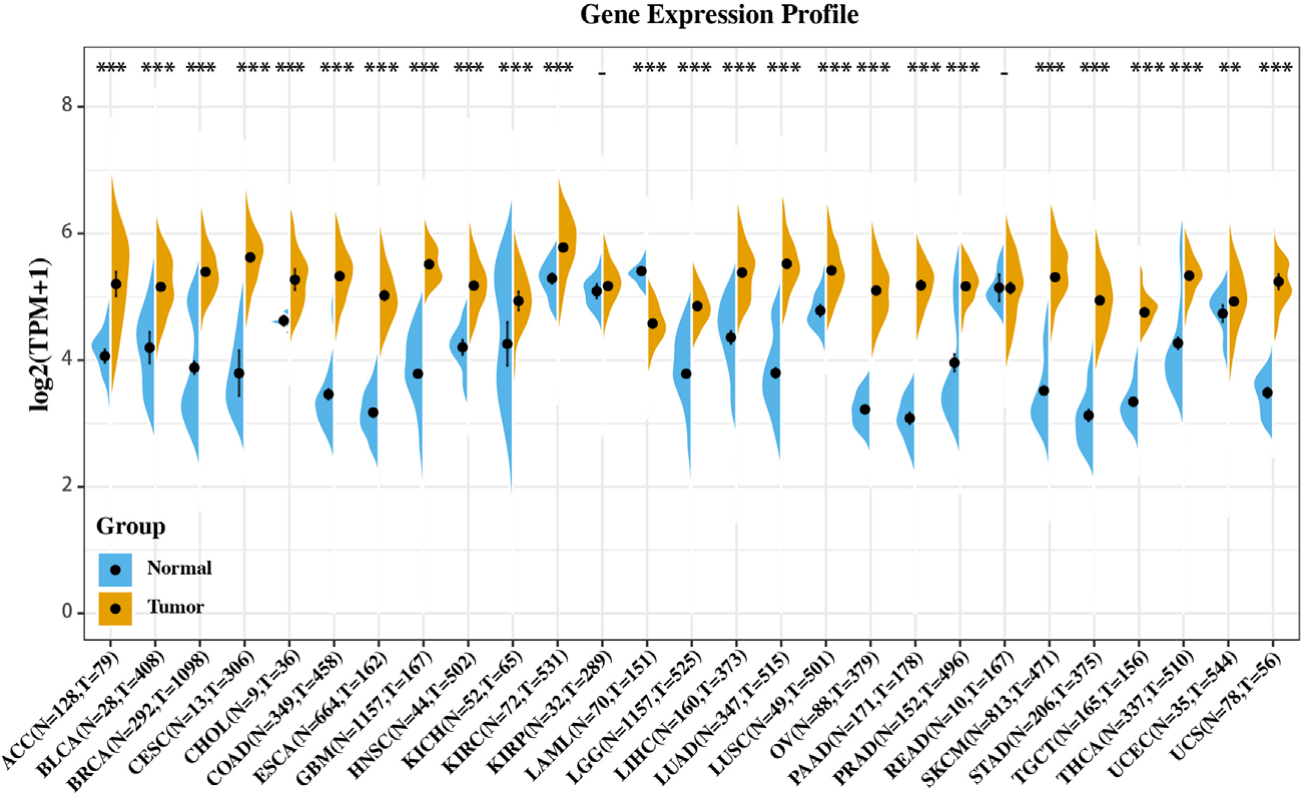
Gene expression profile of CISD2 in 27 types of tumors and its homologous normal tissues. Data was originated from GTEx database and TCGA database and analyzed through Sangerbox platform (http://sangerbox.com). **p < 0.01, ***p < 0.001.

We investigated whether CISD2 is associated with the molecular biological characteristics of cancer. This was done by assessing the expression of CISD2 in 27 tumors and homologous normal samples using the SangerBOX online website based on the samples from The Cancer Genome Atlas (TCGA) and GTEx projects. As shown in **Figure 1**, CISD2 was significantly upregulated in most tumors compared with respectively normal tissues, except for acute myeloid leukemia (LAML), kidney renal papillary cell carcinoma (KIRP), and rectum adenocarcinoma (READ), which indicates that CISD2 might play a critical role in the process of tumorigenesis in various types of cancer.

### High CISD2 Expression Is Linked to High Risk and Poor Prognosis

**Figure 2:**
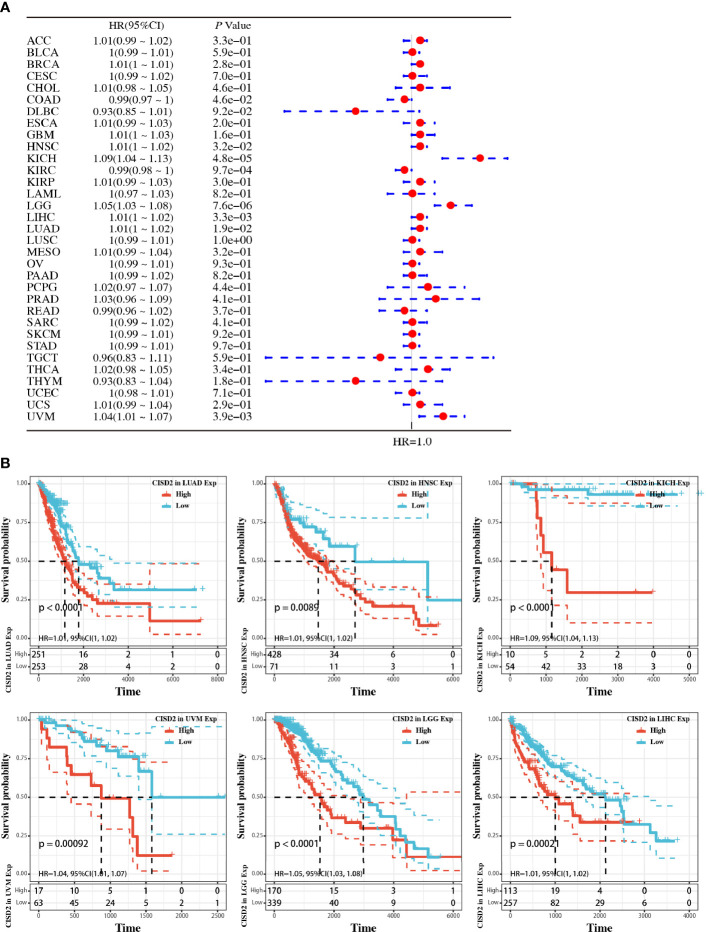
Pan-cancer analysis of the relationship between CISD2 expression and overall survival in 33 TCGA tumors. **(A)** The hazard ratio for overall survival of CISD2 in 33 tumors; the forest diagram was showed in the right. **(B)** Overall survival curves in LUAD, HNSC, KICH, UVM, LGG, and LIHC, respectively.

We additionally employed the prognostic value of CISD2 in cancers within the RNA sequencing data in TCGA to assess how CISD2 expression relates to prognosis in a range of cancer types. The gene expression profile data revealed that CISD2 expression represents a high hazard ratio (HR) in the OS of multiple cancer types (**Figure 2A**). Further data indicated a significantly poorer OS in patients with high CISD2 expression in LUAD, uveal melanoma (UVM), head and neck squamous cell carcinoma (HNSC), brain lower grade glioma (LGG), kidney chromophobe (KICH), and liver hepatocellular carcinoma (LIHC) (**Figure 2B**). These results demonstrated that CISD2 expression might act as a potential indicator of tumor prognosis.

### CISD2 Is Highly Expressed in Lung Cancer

**Figure 3:**
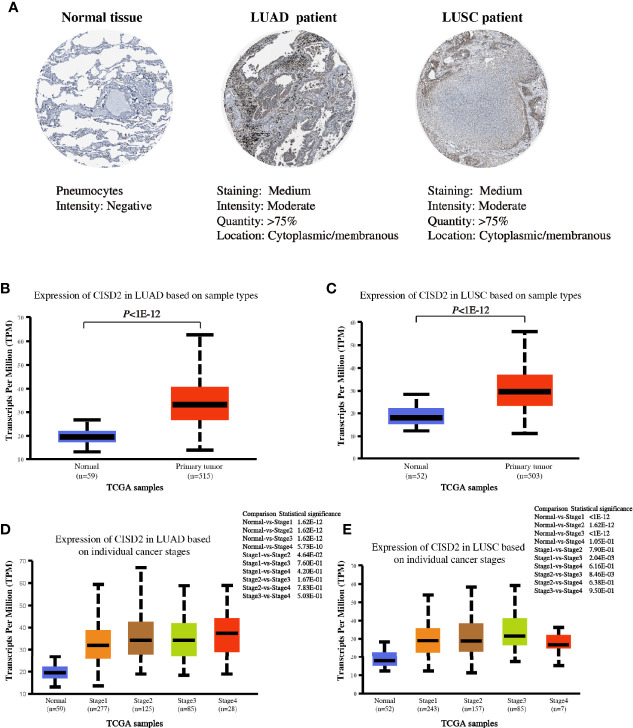
CISD2 expression in lung samples. **(A)** Representative IHC images of CISD2 expression in normal lung tissue, LUAD and LUSC tissues. Data was obtained from The Human Protein Atlas website. **(B)** The expression of CISD2 in LUAD tissues (N = 515) compared with the adjacent normal controls (N = 59). **(C)** The expression of CISD2 in LUSC tissues (N = 503) compared with the adjacent normal controls (N = 52). **(D, E)** CISD2 expression in LUAD or LUSC samples based on the individual cancer stage. These box plots of CISD2 expression originated from TCGA database and analyzed by UALCAN website.

To better characterize the expression of CISD2 in lung tumors, we investigated the expression of this protein in tissues from The Human Protein Atlas database. The data indicated a strong positive cytoplasmic signal of CISD2 protein in lung tumors (LUAD and LUSC) compared with that in normal tissue (**Figure 3A**). We also analyzed the expression of CISD2 in different stages of LUAD and LUSC using the UALCAN website based on the data from TCGA. The results showed an obviously increased CISD2 expression in LUAD and LUSC (**Figures 3B, C**). Subgroup analysis of CISD2 expression was performed in different clinical-pathological stages of lung cancer. The results showed that the expression of CISD2 was significantly higher in individual cancer stages compared with adjacent normal tissues, but not linearly related to pathological stages of LUAD or LUSC (**Figures 3D, E**).

### Increased CISD2 Expression in LUAD Is Linked to Poor Prognosis

**Figure 4:**
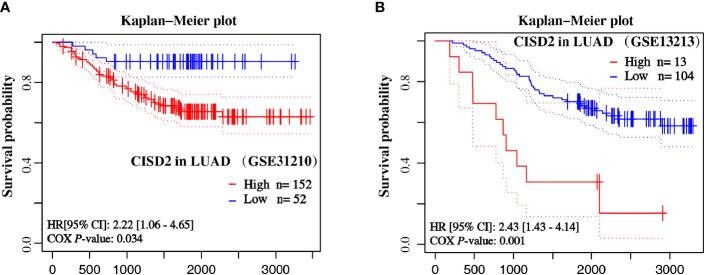
Overall survival curves of LUAD samples based on the expression of CISD2. The Kaplan-Meier plot shows the overall survival of LUAD samples from GSE31210 **(A)** and GSE13213 **(B)** datasets by CISD2 expression.

Moreover, to further verify the association between CISD2 mRNA expression and prognostic outcomes in patients with LUAD, we analyzed two datasets of LUAD (GSE31210 and GSE13213) from the NCBI Gene Expression Omnibus (GEO) datasets. Kaplan-Meier curves of OS were generated from the PrognoScan database using the survival data of the GEO. As shown in **Figure 4**, high CISD2 expression was associated with unfavorable survival in patients with LUAD in the dataset cohorts. The HR of CISD2 expression in GSE31210 and GSE13213 was 2.22 and 2.43, respectively. These results revealed that CISD2 might be associated with poorer outcomes of LUAD.

### CISD2 Silencing Inhibits Viability and Proliferation in LUAD Cells

**Figure 5:**
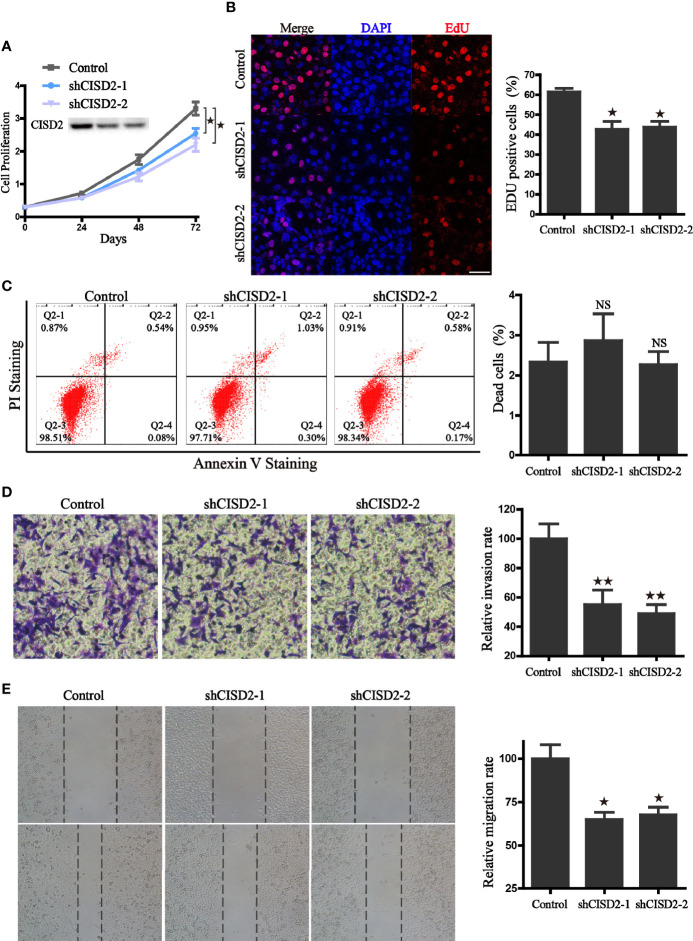
CISD2 associates with the proliferation, invasion, and migration ability of A549 cells. **(A)** The effects of CISD2 knockdown on proliferation rate was evaluated by CCK8 assay, and CISD2 expression was confirmed by western blot. **(B)** Representative fluorescence images (left) of Edu incorporation assay and the statistical graphs (right); DAPI was used for nuclear staining, scale bar: 20 μm. *P < 0.05, compared to the control group. **(C)** Representative images (left) of flow cytometry and the statistical graphs (right) for apoptosis assay; NS, no statistical significance. **(D)** The effects of CISD2 knockdown on cell invasion were evaluated by transwell assay, representative images (left) and the statistical graphs (right) were shown; **P < 0.01, compared to the control group. **(E)** The effects of CISD2 knockdown on cell migration was evaluated by wound-healing assay, representative images (left) and the statistical graphs (right) were shown; *P < 0.05, compared to the control group. Comparable results were obtained in three independent experiments.

To further evaluate the function of CISD2, stable knockdown cells were generated in A549 cells through lentivirus transduction by short hairpin RNA sequences mediated by RNA interference. The effects of CISD2 silencing were validated by western blot (**Figure 5A**). As expected, the knockdown approach efficiently reduced the protein expression of CISD2 in A549 cells. Using these cells, we found that CISD2 depletion did not lead to cell death, but instead inhibited proliferation (**Figures 5A–C**). Compared with the control cells, CISD2 knockdown significantly inhibited the cell viability and proliferation rate of A549 cells. In addition, the EdU incorporation assay revealed that, compared with the control cells, inhibition of CISD2 markedly decreased the divisions of the nucleus in A549 cells (**Figure 5B**). Furthermore, transwell and wound−healing assays were conducted to examine whether the inhibition of CISD2 had an effect on cell migration. The results showed that, compared with the control group, CISD2 silencing significantly inhibited the invasion and migration of cells (**Figures 5D, E**). Taken together, these results suggest that the inhibition of CISD2 suppressed the proliferation and invasion ability of A549 cells.

### CISD2 Is Vital for Maintaining Redox Homeostasis and Mitochondrial Function in LUAD Cells

**Figure 6:**
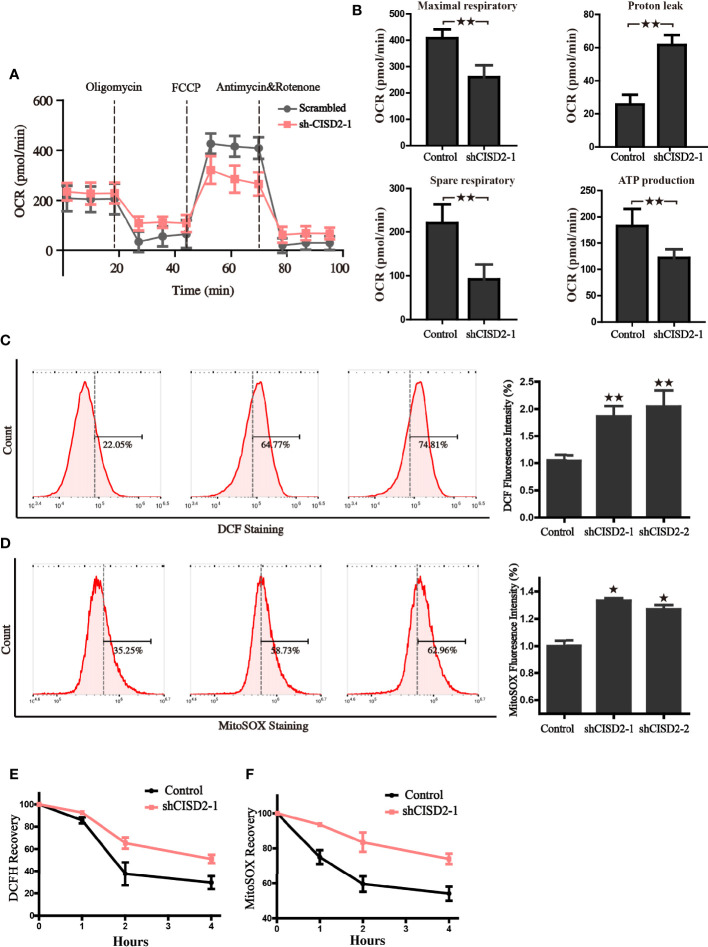
CISD2 is vital for maintaining redox homeostasis and the mitochondrial function in LUAD cells. **(A)** The oxygen consumption rate (OCR) of control and CISD2 silenced cells were measured in real time using the Seahorse XF24 Analyzer. Basal OCR was measured at three time points, following sequential injection of oligomycin (1.5 μM), FCCP (1.5 μM), rotenone (0.5 μM), and antimycin A (0.5 μM). The overall OCR curves were plotted as the mean OCR ± SD of three replicates. **(B)** Maximal respiration, spare respiration, the level of proton leak, and ATP production were calculated according OCR curves, respectively. Data are presented as the mean ± SD, **P < 0.01. **(C)** Cellular ROS levels were detected by flow cytometry in CISD2 knockdown cells. Representative images (left) and statistical graphs (right) are shown; **P < 0.01, compared to the control group. **(D)** Mitochondrial ROS levels were detected by flow cytometry in CISD2 knockdown cells. Representative images (left) and statistical graphs (right) are shown; *P < 0.05, compared to the control group. After treatment of 0.5 mM tBH for 15 min, cells were washed and incubated with complete culture medium (0 h); cellular ROS **(E)** and MitoSOX **(F)** were detected in the indicated time. Comparable results were obtained in three independent experiments.

Given that CISD2 is mainly located in mitochondria, and the Fe-S cluster is closely linked with redox homeostasis regulation and mitochondrial function, we then tested the impact of CISD2 on mitochondrial oxygen consumption and intracellular redox state. The results from seahorse analyzer showed that, compared to the control group, silence of CISD2 hindered the capacity of maximum oxygen respiration, spare respiration, reduced ATP production, and accelerated the level of proton leak (**Figures 6A, B**), which indicates the dysfunction of mitochondria in CISD2-silenced cells. Because of the important role of mitochondria in cellular redox homeostasis, we hypothesized that the decrease of CISD2 expression might give rise to oxidative stress. To verify this hypothesis, the cellular ROS level was examined by DCF-DA staining and flow cytometry, the results showed a significant increase of cellular ROS in CISD2 silenced A549 cells (**Figure 6C**). We further examined the levels of mitochondrial ROS to evaluate the involvement of mitochondria in the tumor inhibitory effects of CISD2 knockdown A549 cells. The flow cytometry analysis of MitoSOX staining showed that CISD2 knockdown led to significant increases in mitochondrial ROS (**Figure 6D**). These data demonstrated that knockdown of CISD2 facilitated the accumulation of peroxide radicals and led to mitochondrial dysfunction. Next, we tested the ability of CISD2 to recover from oxygen stress in the transitory treatment of *tert*-butylhydroquinone (tBH). A time-course of the mitoSOX and DCF fluorescence was monitored, and results showed that CISD2 knockdown hindered the oxygen stress recovery ability (**Figures 6E, F**). The above data demonstrate that CISD2 is indispensable for resisting oxidative stress and maintaining the mitochondrial function in A549 cells.

### CISD2 Knockdown Accelerates Iron Accumulation in LUAD Cells

**Figure 7:**
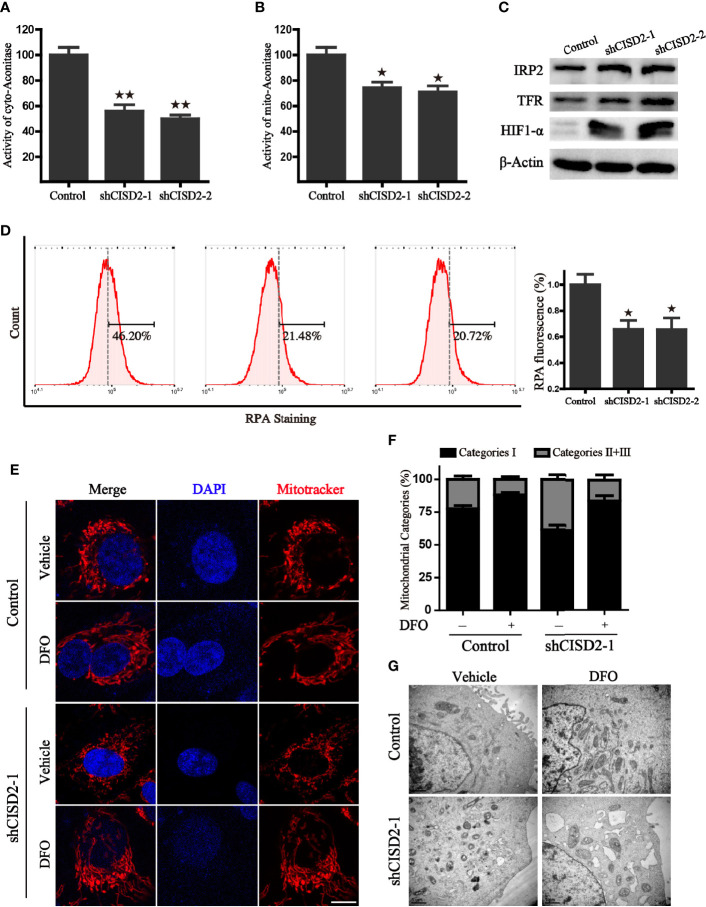
CISD2 silencing accelerates iron accumulation in A549 cells. **(A)** The activity of cytoplasmic aconitase in CISD2 knockdown cells, **P < 0.01 *vs.* the control group. **(B)** The activity of mitochondrial aconitase in CISD2 knockdown cells, *P < 0.05 *vs.* the control group. **(C)** Protein levels of iron regulatory protein 2 (IRP2), transferrin receptor protein (TFR), and hypoxia-inducible factor 1-alpha (HIF1-α) in indicated A549 cells. β-Actin was used as the loading control. **(D)** Intracellular Fe^2+^ was measured by RPA staining and flow cytometry analysis. Representative images (left) and statistical graphs (right) are shown; *P < 0.05, compared to the control group. **(E)** The representative images of mitochondrial morphology. Control and CISD2 silenced A549 cells were treated with or without DFO (100 μM) for 12 h, then labeled with MitoTracker Red and subjected to a confocal microscope for the observation of changes in mitochondrial morphology. Scale bars: 5 µm. **(F)** Quantification of mitochondrial morphology alteration according to the classification of mitochondrial category I–III. **(G)** The morphological changes of mitochondria in control and CISD2 silenced cells were detected by transmission electron microscopy (TEM) in the presence or absence of DFO (100 μM) treatment. Comparable results were obtained in three independent experiments.

Given that the Fe-S cluster participates in the regulation of iron metabolism, we investigated whether the alteration of CISD2 expression gives rise to iron-starving stress. We first tested the activity of mitochondrial and cytoplasmic aconitase, which are important [4Fe- 4S] proteins that catalyze the reaction from citric acid to isocitrate and rely on their intact iron-sulfur clusters. The results revealed that CISD2 knockdown resulted in a more dramatic decrease of cytoplasmic aconitase activity than m-aconitase activity, demonstrating that CISD2 depletion promoted the loss of aconitase ISCs (**Figures 7A, B**). It has previously been reported that cellular iron-responsive protein 1 (IRP1) and IRP2 play a central role in the regulation of iron metabolism. The apo form of cytoplasmic aconitase transforms into IRP1, which could promote the degradation of the ferritin mRNA and stabilization of transferrin receptor 1 (TFR1) mRNA through the IRP-IRE mechanism, leading to the iron starvation response. In line with reduced aconitase activity, we found that CISD2 silencing induced an increase of IRP2 and TFR, further indicating the activation of iron starving stress in cells (**Figure 7C**). We next used the selective yield fluorescence probe RPA, whose cationic fluorophore is rapidly quenched by Fe^2+^ ions, to assess the cellular labile iron pool. The fluorescence of RPA was significantly decreased in the CISD2 knockdown A549 cells with the increased intracellular iron level (**Figure 7D**), suggesting that CISD2 plays a role in maintaining iron homeostasis, which is consistent with the results of a previous study (6). Moreover, DFO, an iron-chelating agent, was used to investigate whether the depletion of iron could block mitochondrial dysfunction in CISD2 knockdown cells. Mitochondria were labeled with the mitotracker probe and visualized with a confocal laser scanning microscope. The CISD2 silenced cells appeared to contain fragmented mitochondria, which accumulated around the nucleus, whereas pretreatment of DFO significantly ameliorated the destruction of mitochondrial morphology, which was shown as a network of elongated mitochondria (**Figure 7E**). To further address mitochondrial alterations, mitochondria were categorized into three classes. Pretreatment of DFO blocked the reduction in the proportion of category I (**Figure 7F**). Furthermore, the change of mitochondrial morphology was also confirmed by transmission electron microscope and the results consistent with the mitotracker staining (**Figure 7G**). These data indicate that the knockdown of CISD2 results in iron-starving stress and destroyed mitochondrial morphology.

## Discussion

At present, an increasing amount of evidence suggests that cancer cells depend on high levels of the iron-sulfur cluster biosynthetic enzyme (8). For instance, Possemato et al. demonstrated that NFS1 suppression resulted in proliferation arrest upon exposure to atmospheric oxygen and predisposed cancer cells to ferroptosis (9). Moreover, the p53-ISCU pathway plays a role in hepatocellular carcinogenesis *via* the regulation of iron homeostasis (10). Our previous studies demonstrate that the Fe-S cluster assembly proteins ISCU and FXN were able to rescue the ferroptotic process by regulating iron homeostasis and mitochondrial function (11, 12). In addition, accumulating evidence suggests that cancer cells often exhibit an enhanced dependence on high iron levels, which take part in processes such as DNA and protein synthesis, glycolysis, and respiration (13, 14). Consequently, treatments that target the iron homeostasis of cancer cells have been proposed as a promising strategy.

CISD2 could represent a key regulatory link between iron metabolism and cellular ROS in cancer cells due to its unique 2Fe-2S cluster coordinated by 3Cys:1His, which is labile and redox-active. Studies by Nechushtai et al. have demonstrated that the Fe-S cluster in CISD2 is central to human breast cancer proliferation by maintaining mitochondrial homeostasis, and could be developed as a fundamental chemotherapeutic target (15, 16). A recent study also showed that CISD2 is significantly upregulated in gastric cancer, and its expression has been significantly associated with the clinical stage, TNM classifications, venous invasion, and lymphatic invasion (17). Despite these findings, the mechanisms underlying its regulation role in the redox state and mitochondrial homeostasis remain unclear.

As CISD2 is a conservative protein, bioinformatics of pan-cancer analysis from TCGA show that the expression of CISD2 is generally increased in 27 types of human cancers, except for LAML, KIRP, and READ, indicating that CISD2 could play a critical role in tumorigenesis. In addition, patients with high CISD2 expression have been linked to a higher HR and significantly poorer OS in LUAD, UVM, HNSC, LGG, KICH, and LIHC, suggesting that high CISD2 expression could act as an indicator of tumor prognosis. Further study found that CISD2 is highly expressed in LUAD and LUSC, which is associated with clinical pathological stages. In addition, survival data collected from GSE31210 and GSE13213, two datasets from the GEO, also confirmed that high CISD2 expression is associated with unfavorable survival in patients with LUAD. Consistent with the bioinformatic analysis, we also generated CISD2 knockdown A549 cells through lentivirus mediated shRNA interference. We found that CISD2 depletion did not lead to cell death, but instead inhibited cellular proliferation, as determined by the cell proliferation rate and EdU incorporation assay. Collectively, we determined that CISD2 plays a role in the molecular biological characteristics of cancer.

Mitochondria are essential for cellular metabolism, signal transduction, and death signals, which determines the fate of cells (18). Once mitochondrial dysfunction occurs, large quantities of ROS, including hydrogen peroxide, superoxide anion, hydroxyl radical, and peroxynitrite, are produced from mitochondrial metabolism and coordinate intracellular signal transduction (19). Previous studies have demonstrated that CISD2 is mainly located in the outer mitochondrial membrane and participates in Fe-S cluster transfer. Holt et al. revealed that CISD2 is a major player in the metabolic regulation of breast cancer cells through its effects on mitochondrial metabolism and the induction of apoptosis (20). Chen et al. demonstrated that CISD2 is crucial for the maintenance of mitochondrial integrity, and its deficiency can cause mitochondrial dysfunction accompanied by the initiation of autophagy (21). In this study, we found that CISD2 knockdown led to increased ROS production and reduced ATP generation, which was unable to meet the cellular ATP demands for supporting cell growth. As mitochondrial morphology is a valuable parameter for the judgment of mitochondrial dysfunction, we also observed that fragmentation mitochondria accumulated around the nucleus in CISD2-depleted cells. Therefore, it is conceivable that CISD2 is a key regulator of mitochondrial homeostasis, and its depletion accelerates ROS generation by inducing mitochondrial dysfunction.

The highly reductive and soluble Fe^2+^ serves as a catalyst and cofactor, participating in various biological processes and constituting the labile/free iron pool. Amplifying cancer cells harbor labile Fe^2+^ mainly in the mitochondria and produce hydroxyl radicals *via* the Fenton reaction (22). Alteration of free iron levels and iron metabolism proteins are involved in the regulation of ferroptosis sensitivity (23). As demonstrated previously, CISD2 inhibition overcomes resistance to SASP-induced ferroptosis through the increased accumulation of mitochondrial ferrous iron and lipid ROS in head and neck cancer (24). Therefore, targeting cellular catalyzed iron metabolism is a promising therapeutic strategy for inducing tumorous ferroptosis. In the present study, inhibition of CISD2 significantly activated iron-starving stress and subsequently increased the labile iron levels with the decreased fluorescence of RPA. The administration of iron chelator was able to rescue the mitochondrial morphology. Therefore, dysfunction of iron metabolism partly contributes to the CISD2-mediated mitochondrial dysfunction.

Overall, we have defined a regulatory signaling pathway mediated by CISD2 in lung cancer that regulates the homeostasis of mitochondria and iron metabolism and may be developed as a novel pharmacological agent.

## Data Availability Statement

The data that support the findings of this study are available from the authors upon reasonable request.

## Author Contributions

JD, JX, KY: Supervision, methodology, manuscript review. YL, FS, JD: Project administration, writing original draft. YL, WH, JY, HW: Data acquisition and analysis, manuscript review. All authors contributed to the article and approved the submitted version.

## Funding

This research was supported by Zhejiang Public Welfare Technology Application Research Project (Grant Nos. LGF19H080006, LGF21H010008, LGF20H080005), Medical and Health Science and Technology Project of Zhejiang Province (Nos. 2019RC014, 2019RC115, 2021KY842, 2021KY483, 2021KY077). The Foundation of Zhejiang Provincial Administration of Traditional Chinese Medicine (No. 2020ZB020).

## Conflict of Interest

The authors declare that the research was conducted in the absence of any commercial or financial relationships that could be construed as a potential conflict of interest.
